# Antiamylase, Antiglucosidase, and Antiglycation Properties of Millets and Sorghum from Sri Lanka

**DOI:** 10.1155/2021/5834915

**Published:** 2021-06-17

**Authors:** Ilangasingha Gamlathge Nethmini Hansika Senevirathne, Walimuni Kanchana Subhashini Mendis Abeysekera, Walimuni Prabhashini Kaushalya Mendis Abeysekera, Nileththi Yasendra Jayanath, Sirimal Premakumara Galbada Arachchige, Danthasingha Chithra Mulacharige Susantha Indika Wijewardana

**Affiliations:** ^1^Department of Food Science and Technology, Faculty of Agriculture, University of Peradeniya, Peradeniya, Kandy, Sri Lanka; ^2^Department of Agricultural Technology, Faculty of Technology, University of Colombo, Colombo, Sri Lanka; ^3^Department of Biosystems Technology, Faculty of Technology, University of Sri Jayewardenepura, Nugegoda, Colombo, Sri Lanka; ^4^Industrial Technology Institute (ITI), Halbarawa Gardens, Malabe, Colombo, Sri Lanka; ^5^Department of Basic Science & Social Science, Faculty of Nursing, University of Colombo, Colombo, Sri Lanka; ^6^Field Crop Research and Development Institute, Department of Agriculture, Mahailuppallama, Sri Lanka

## Abstract

The present study evaluated a range of biological activities of selected millet types and sorghum varieties in Sri Lanka in relation to diabetes and its complications management. Five millet types, namely, proso millet, white finger millet, kodo millet, foxtail millet, and finger millet (Oshadha and Rawana), and two sorghum varieties, namely, sweet sorghum and sorghum ICSV 112, were used in this study. Methanolic extracts of whole grains were studied for antiamylase, antiglucosidase, and early- and middle-stage antiglycation and glycation reversing activities *in vitro*. Tested millets and sorghum showed significant (*p* < 0.05) and dose-dependent antiamylase (IC_50_: 33.34 ± 1.11–1446.70 ± 54.10 *μ*g/ml), early-stage antiglycation (IC_50_: 15.42 ± 0.50–270.03 ± 16.29 *μ*g/ml), middle-stage antiglycation (135.08 ± 12.95–614.54 ± 6.99 *μ*g/ml), early-stage glycation reversing (EC_50_: 91.82 ± 6.56–783.20 ± 61.70 *μ*g/ml), and middle-stage glycation reversing (393.24 ± 8.68–1374.60 ± 129.30 *μ*g/ml) activities. However, none of the studied millet and sorghum showed antiglucosidase activity. Out of the samples studied, pigmented samples, namely, sweet sorghum, Oshadha, and Rawana, exhibited significantly high (*p* < 0.05) antiamylase and early- and middle-stage antiglycation and glycation reversing activities compared to other millet and sorghum samples. Interestingly, sweet sorghum exhibited nearly four times potent antiamylase activity compared to the standard drug acarbose (IC_50_ 111.98 ± 2.68 *μ*g/ml) and sweet sorghum, kodo millet, Oshadha, and Rawana showed comparable early-stage antiglycation activities in comparison to the reference standard Rutin (IC_50_ 21.88 ± 0.16 *μ*g/ml). Therefore, consumption of whole grains of pigmented millet and sorghum in Sri Lanka may play an important role in the prevention and management of diabetes and its complications. Interestingly, this is the 1^st^ study to report all the tested biological activities for millet and sorghum in Sri Lanka and the 1^st^ study to report both early- and middle-stage glycation reversing activities of millet and sorghum worldwide.

## 1. Introduction

Non-communicable diseases (NCDs) which are also known as the chronic diseases or lifestyle-related diseases are the world's leading causes of deaths [1]. Diabetes mellitus is among the top four major NCDs word-over [2]. According to the most recent health statistics by the International Diabetes Federation 2019, approximately 463 million people worldwide have diabetes and this number is projected to increase by 700 million by 2045 [3]. Notably, around 75% of diabetes patients in the world live in low and middle income countries [3].

Diabetes is characterized by hyperglycaemia and long-term hyperglycaemic conditions in diabetes patients lead to diabetes retinopathy, nephropathy, neuropathy, increased risk of cancers, cardiovascular diseases, rheumatic arthritis, osteoarthritis and neurodegenerative diseases such as Alzheimer's disease, Parkinson disease, and age-related cognitive decline [4–8]. Numerous research findings have clearly shown that these diseases and complications have a promising link with the glycated proteins and Advanced Glycation End Products (AGEs), which are formed through the protein glycation reaction under hyperglycaemic conditions in diabetes patients [7]. However, protein glycation is not a simple reaction and is defined as a series of complex non-enzymatic reactions between proteins and sugars giving rise to a multitude of heterogeneous glycated end products which are widely known as the AGEs [9, 10]. Glycation reaction is broadly categorized into 3 stages as early-, middle-, and late-stage glycation [7, 11]. At the early stage of protein glycation process, reaction between carbonyl groups of reducing sugars and amino group of proteins leads to the production of Amadori compounds. In the middle stage, reactive dicarbonyl compounds such as methylglyoxal (MGO), glyoxal, and glucosone are produced via auto oxidation of glucose and glycoxidation of Amadori compounds while at the last stage dicarbonyl compounds undergo further glycoxidation to form AGEs and their subsequent cross-linked glycated products [7, 11]. Therefore, in the prevention and management of diabetes and its complications, the compounds which can reduce the hyperglycaemia as well as the glycation inhibitors, among which most importantly compounds that can reverse the glycated proteins are of immensely valuable.

Natural products are widely known for array of health benefits including antidiabetic properties worldwide [11–14]. Millets and sorghum are small seeded cereal crops which are dietary staples for millions of people in developing countries especially in the semiarid tropics of Asia and Africa [15, 16]. Different types of millets have been identified namely foxtail millet *(Setaria italica)*, finger millet *(Eleusine coracana)*, proso millet *(Panicum miliaceum)*, and kodo millet *(Paspalum scrobiculatum)* while widely cultivating sorghum in the world is known as the *Sorghum bicolor*. Sorghum and millet are ranked in the world cereal production 5^th^ and 6^th^ places, respectively [16]. Whole grain millets and sorghum contain significant amounts of phytochemicals which are responsible for wide variety of health benefits such as antiproliferative, antimicrobial, anticancer, antilipidemic, anti-inflammatory, and some antidiabetic properties worldwide [15, 17, 18]. In Sri Lanka also, millet and sorghum are important cereals and different types of pigmented and nonpigmented millet types and sorghum varieties are currently cultivated island wide [19]. However, to date except some antioxidant [19–21] and antiamylase [22] activities other biological activities of millet and sorghum varieties in Sri Lanka are not documented. To the best of our knowledge, glycation reversing activities of both millet and sorghum varieties in the world are not yet reported. Further, extremely limited studies are reported on antiamylase and antiglycation activities of millet types and sorghum varieties world-over. In this study, we report antiamylase, antiglucosidase, and early- and middle-stage antiglycation and glycation reversing activities of range of millet types and sorghum varieties in Sri Lanka.

## 2. Materials and Methods

### 2.1. Chemicals and Reagents

Bovine Serum Albumin (BSA), D-glucose, soluble starch, *α*-glucosidase (type V from rice), *α*-amylase (*Bacillus amyloliquefaciens*), p-nitrophenyl *α*-D-glucopyranoside, acarbose, methylglyoxal, Rutin, trichloro acetic acid (TCA), 3,5dinitrosalicylic acid (DNS), and dimethyl sulfoxide (DMSO) were purchased from Sigma-Aldrich, St Louis, USA. All the other chemicals and reagents used were of analytical grade.

### 2.2. Sample Collection

**Figure 1:**
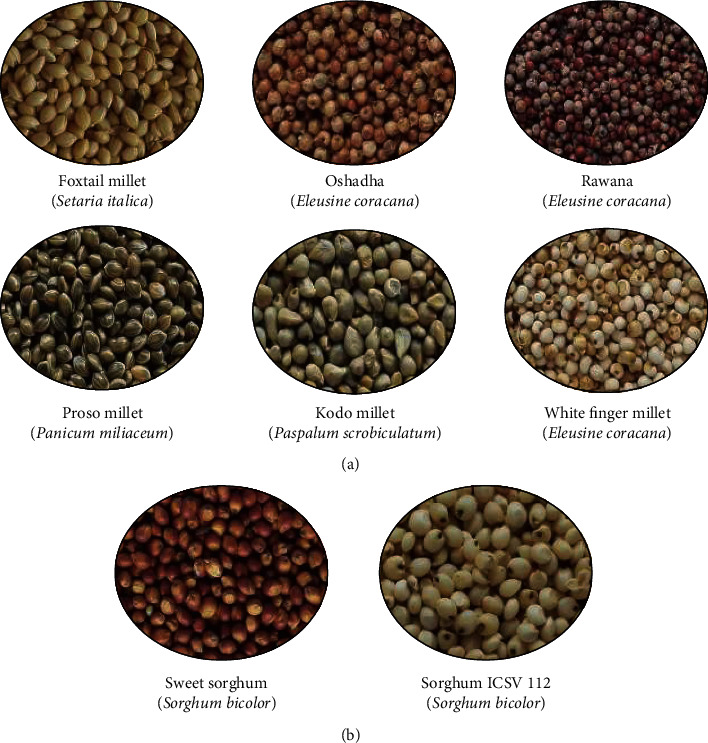
Millet types and sorghum varieties selected. (a) Millet types. (b) Sorghum varieties.

Five millet types, namely, foxtail millet *(Setaria italica)*, finger millet *(Eleusine coracana)* [Oshadha & Rawana varieties], proso millet *(Panicum miliaceum)*, kodo millet *(Paspalum scrobiculatum)*, and white finger millet *(Eleusine coracana)*, and two sorghum *(Sorghum bicolor)* varieties, namely, sweet sorghum and sorghum ICSV 112 (see Figure 1), were collected from Field Crop Research and Development Institute, Mahailluppallama, Sri Lanka.

### 2.3. Preparation of Samples

Whole grains of selected samples were milled using a laboratory mill (Fritsch, Pulverisette 14, Germany) and passed through a 0.5 mm sieve to obtain flour. Then, whole grain flour of each millet type and sorghum varieties were kept at 4°C in a laboratory refrigerator until use for the analysis.

### 2.4. Preparation of Extracts

One gram of flour from selected millet types and sorghum varieties was extracted in 100 ml of methanol for overnight at 160 rpm using a laboratory shaker at room temperature (28 ± 2°C). Extracts were then centrifuged (825 g), filtered and evaporated under vacuum using a rotary evaporator, and freeze-dried (Christ-Alpha 1–4 Freeze dryer, Biotech International, Germany).

### 2.5. Enzyme Inhibitory Activity

#### 2.5.1. Antiamylase Activity

Antiamylase activity of selected samples was determined according to the methods of Bernfeld and Premakumara et al. [23, 24] with some modifications using 96-well micro plates. A reaction volume of 1 ml containing 40 *μ*l of starch (1% w/v), 60 *μ*l of 5 *μ*g/ml *α*–amylase (*Bacillus amyloliquefaciens*), 50 *μ*l of millet and sorghum (1 mg/ml) samples, and 850 *μ*l of 100 mM sodium acetate buffer (pH 6.0) were incubated at 40°C for 10 min. Then, 500 *μ*l of DNS reagent was added and boiled for 8 min. Samples were then allowed to cool in an ice bath and the absorbance readings were taken at 540 nm using a 96-well micro plate reader (SpectraMax Plus^384^, Molecular Devices, USA). For dose response studies, the samples which showed the highest inhibitory activities at screening were selected and antiamylase activity was studied using a series of concentrations (62.5, 125, 250, 500, and 1000 *μ*g/ml; *n* = 3). Acarbose (6.25, 12.5, 25, 50, 100 *μ*g/ml) was used as the positive control. Results were expressed as % antiamylase activity and IC_50_ values.

#### 2.5.2. Antiglucosidase Activity

Antiglucosidase activity of selected samples was determined according to the methods of Premakumara et al. and Matsui et al. [24, 25] with minor modifications using 96-well microplates. A reaction volume of 100 *μ*l containing 10 *μ*l of millet and sorghum samples (1 mg/ml), 30 *μ*l of 50 mU/ml *α*–glucosidase (type V from rice), 10 *μ*l of acetate buffer, and 50 *μ*l of p-nitrophenyl-*α*-D glucopyranoside (6 mg/ml) were incubated at 37°C for 30 min. Then, 50 *μ*l of 10% Na_2_CO_3_ was added and the absorbance readings were recorded at 405 nm using a 96-well microplate reader (SpectraMax Plus^384^, Molecular Devices, USA). Acarbose (0.125, 0.25, 0.5, 1 *μ*g/ml) was used as the positive control. Results were presented as % antiglucosidase activity.

### 2.6. Diabetes Complication Management

#### 2.6.1. Early-Stage Antiglycation Activity

BSA-glucose mediated early-stage antiglycation activity of selected millet types and sorghum varieties was determined according to the methods of Matsuura et al., Ratnasooriya et al., and Arachchige et al. [26–28] with some modifications. A reaction volume of 1 ml containing 10 mg/ml BSA, 200 mg/ml glucose, and different concentrations of millet and sorghum samples (12.5, 25, 50, 100, 200 *μ*g/ml; *n* = 4) in 50 mM phosphate buffer (pH 7.4) containing 0.02% sodium azide were incubated at 60°C for 40 h. Then, 600 *μ*l of each reaction mixture was transferred to Eppendorf tubes and 120 *μ*l of 50% (w/v) TCA was added and kept at room temperature (25 ± 2°C) for 20 min. Then, samples were centrifuged at 15 000 rpm for 4 min at 4°C and the supernatants were removed. The resultant precipitates were dissolved in 1 ml of phosphate buffer saline (pH 10) and the fluorescence intensities were measured at an excitation and emission wave lengths of 370 nm and 440 nm, respectively, using a florescence 96-well microplate reader (SpectraMax, Gemini EM, Molecular Devices, Inc., USA). Rutin (6.25, 12.5, 25, 50, 100 *μ*g/ml) was used as the positive control. Results were expressed as % early-stage antiglycation activity and IC_50_ values.

#### 2.6.2. Early-Stage Glycation Reversing Activity

BSA-glucose mediated early-stage glycation reversing activity of selected samples was determined according to the methods of Premakumara et al., Arachchige et al., and Abeysekera et al. [24, 28, 29] with some modifications. Glycated proteins were prepared as described in the early-stage antiglycation activity (reaction mixture without addition of samples; see Section 2.6.1). Then, glycated proteins were dissolved in 0.1 M phosphate buffer (pH 7.4) and a reaction volume of 1 ml containing different concentrations of millet and sorghum samples (100, 200, 400 *μ*g/ml; *n* = 3) was incubated at 60°C for 40 h. Reaction mixture without addition of samples was regarded as the control. After the incubation period, 120 *μ*l of TCA (w/v) was added, allowed to stand at room temperature (25 ± 2°C) for 20 min, and centrifuged (15,000 rpm, 4 min, 4°C), supernatants were discarded, and the precipitates were dissolved in 1 ml phosphate buffer saline (pH 7.4). The fluorescence intensity was measured at excitation and emission wave lengths of 370 and 440 nm, respectively, using a florescence 96-well microplate reader (SpectraMax, Gemini EM, Molecular Devices, Inc., USA). Results were expressed as % early-stage glycation reversing activity and EC_50_ values.

#### 2.6.3. Middle-Stage Antiglycation Activity

BSA-MGO mediated middle-stage antiglycation activity of the selected millet and sorghum samples was determined according to the methods of Abeysekera et al. and Lunceford and Gugliucci [30, 31] with some modifications. A reaction volume of 1 ml containing 10 mg/ml BSA, 5 mM MGO, and different concentrations of millet and sorghum samples (50, 100, 200, 400 *μ*g/ml; *n* = 4 each) in 0.1 M phosphate buffer (pH 7.4) containing 0.02% sodium azide were incubated at 37°C for 6 days. Then, fluorescence intensity was measured at an excitation and emission wave lengths of 370 nm and 440 nm, respectively, using a florescence 96-well microplate reader (SpectraMax, Gemini EM, Molecular Devices, Inc., USA). Rutin (6.25, 12.5, 25, 50, 100, 200 *μ*g/ml) was used as the positive control. Results were expressed as % middle-stage antiglycation activity and IC_50_ values.

#### 2.6.4. Middle-Stage Glycation Reversing Activity

BSA-MGO mediated middle-stage glycation reversing activity was determined according to the methods of Ratnasooriya et al. and Arachchige et al. [27, 28] with some modifications. The millet and sorghum samples which showed the highest early-stage glycation reversing activity via BSA-Glucose model were used in this experiment. Glycated proteins were prepared as described in the middle-stage antiglycation activity (reaction mixture without addition of samples; see Section 2.6.3). Then, glycated proteins were dissolved in 0.1 M phosphate buffer (pH 7.4) and a reaction volume of 1 ml containing different concentrations of millet and sorghum samples (125, 250, 500, 1000 *μ*g/ml; *n* = 4) was incubated at 37°C for 6 days. Reaction mixture without addition of samples was regarded as the control. The fluorescence intensity was measured at excitation and emission wave lengths of 370 nm and 440 nm, respectively, using a florescence 96-well microplate reader (SpectraMax, Gemini EM, Molecular Devices, Inc., USA). Results were expressed as % middle-stage glycation reversing activity and EC_50_ values.

### 2.7. Statistical Analysis

Data were statistically analysed using Minitab software (Version 17.3.1, Minitab, Inc, Pennsylvania, USA). Experiments were carried out in triplicates (*n* = 3) and results were expressed as mean ± standard deviation (SD). The differences of mean values among samples were determined by one-way analysis of variance (ANOVA) followed by Turkey's Honestly Significant Difference (HSD) multiple rank tests at *p* ≤ 0.05 significance level.

## 3. Results and Discussion

### 3.1. Enzyme Inhibitory Activity

#### 3.1.1. Antiamylase Activity

**Figure 2:**
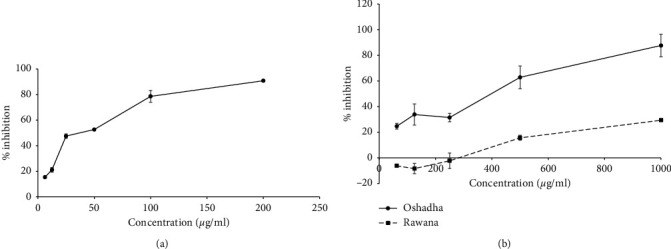
Dose response relationship of tested samples for antiamylase activity. (a) Dose response relationship of sweet sorghum. (b) Dose response relationship of Oshadha and Rawana.

*α*-Amylase is identified as a key enzyme which is involved in carbohydrate digestion and inhibition of this enzyme is known to play a vital role in regulation of blood glucose level [32]. Percent antiamylase activity of the selected millet types and sorghum varieties were screened at 1 mg/ml concentration. All selected millet types and sorghum varieties in Sri Lanka showed % antiamylase activity in varying degrees of potentials ranging from 11.07 ± 0.32% to 100.00 ± 0.29%. Sweet sorghum exhibited the highest inhibitory activity among the samples screened at the tested concentration (100% inhibitory activity). Samples which showed the highest inhibitory activities at screening were further investigated for antiamylase activity using a series of concentrations to study the dose response relationship and results are given in Figures 2(a) and 2(b) and Table 1 (IC_50_ values). The three studied samples for dose response relationship, namely, sweet sorghum (*r*^2^ = 0.97), Oshadha (*r*^2^ = 0.96), and Rawana (*r*^2^ = 0.95), exhibited significant (*p* < 0.05) and dose-dependent relationship and the IC_50_ values were 33.34 ± 1.11, 430.90 ± 33.50, and 1446.70 ± 54.10 *μ*g/ml, respectively (see Table 1). The order of potency of three samples for antiamylase activity was sweet sorghum > Oshadha > Rawana. Interestingly, sweet sorghum exhibited nearly 4 times potent antiamylase activity compared to the reference drug acarbose (IC_50_ 111.98 ± 2.68 *μ*g/ml) used in this study.

There are limited number of studies on antiamylase activity of millet and sorghum world-over [33–35]. A study by Hargrove et al. [33] reported that proanthocyanidin rich sumac sorghum bran extracts could inhibit *α*-amylase enzyme at low concentrations (IC_50_: 1.4 *μ*g of phenolics/ml). Antiamylase activity of foxtail millet studied by Pradeep and Sreerama [34] has reported that highest inhibitory activities were in the hull fractions and the IC_50_ values of soluble and bound extracts were 32.29 and 41.74 *μ*g ferulic acid equivalents/ml, respectively. A very recent study conducted by Ofosu et al. [35] showed that pigmented millet varieties had the highest antiamylase activity compared to the nonpigmented millet samples and the IC_50_ values were in the range of 10.56 ± 1.43–81.32 ± 3.54 *μ*g/ml. In this study, we also observed that pigmented millet and sorghum varieties of Sri Lanka had the highest antiamylase activity and our findings are in accordance with the previous research findings. It is interesting to highlight that sweet sorghum tested in the present study had potent antiamylase activity compared to the other millet and sorghum varieties world over and it is far superior (4 times) to the clinical drug acarbose. This might be due to the genotype and differences in the extraction protocols. Therefore, in future studies it is important to perform activity guided separation to find novel *α*-amylase inhibitors from sweet sorghum cultivated in Sri Lanka.

Pigmented millet and sorghum are rich sources of phenolic antioxidants [15]. These phenolics are reported to involve in inhibition of *α*-amylase enzyme thus important in managing hyperglycemic conditions [17]. Quantification of polyphenolic compounds in millet types in Sri Lanka has been studied in the past few years [19–22]. According to the findings of these studies, kodo millet and finger millets have shown high contents of phenolic antioxidants while proso millet had the lowest. We recently reported total proanthocyanidin content of millet types and sorghum varieties in Sri Lanka and interestingly sweet sorghum, Oshadha, and Rawana showed the greatest total proanthocyanidin content [36]. Therefore, the observed antiamylase activities of selected millet types and sorghum varieties in Sri Lanka might be due to the presence of polyphenolic antioxidants and proanthocyanidins.

#### 3.1.2. Antiglucosidase Activity

*α*-Glucosidases are brush border enzymes involved in carbohydrate digestion. Inhibitors of this enzymes slow down the carbohydrate digestion thereby reducing the elevation of blood sugar level following a carbohydrate meal [17]. Results of the present study showed that none of the selected millet types and sorghum samples had antiglucosidase activity at the tested concentration (1 mg/ml). The concentrations higher than the tested concentration were not evaluated in the present study as pigments in millet and sorghum samples interfere with the assay condition. Further, the tested concentration is an agreeable concentration for a natural product to show a biological activity of interest. Few studies on antiglucosidase activity of millet types and sorghum varieties have reported the presence of antiglucosidase activity in germinated, nongerminated, whole grains, and isolated compounds of millet and sorghum [34, 35, 37, 38]. In these studies, the antiglucosidase activity of millet and sorghum has shown wide variation. This may be due to varietal differences, processing conditions, variation in extraction protocols, changes in the source of enzyme, and the differences in the assay conditions. The absence of antiglucosidase activity of selected millet types and sorghum varieties in Sri Lanka at tested concentration in the present study also may be due to the above stated factors.

### 3.2. Diabetes Complication Management

#### 3.2.1. Early-Stage Antiglycation and Glycation Reversing Activities

**Figure 3:**
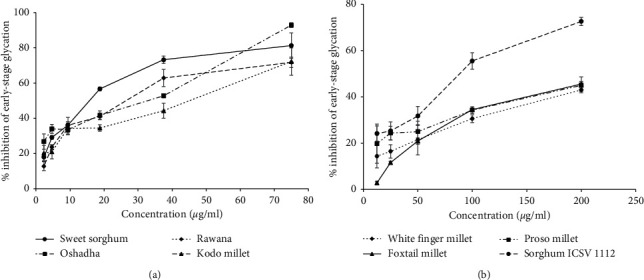
Early-stage antiglycation activity of samples. (a) Dose response relationship of sweet sorghum, kodo millet, Oshadha, and Rawana. (b) Dose response relationship of sorghum ICSV 112, proso millet, white finger millet, and foxtail millet.

Long-term hyperglycemic conditions in diabetic patients cause formation of glycated proteins through the protein glycation reaction. Initial stages of this reaction begin due to the action of reducing sugars and proteins [7, 14]. Thus, the early stage of protein glycation in this study was evaluated via BSA-glucose model. BSA was selected as it resembles to albumin and glucose was selected as it is the most widely available sugar in the human body [39]. Therefore, *in vitro* conditions selected in the present study mimic *in vivo* conditions. The dose response relationship of early-stage antiglycation activity of selected millet types and sorghum varieties in Sri Lanka is given in Figures 3(a) and 3(b). All the eight samples, namely, sweet sorghum (*r*^2^ = 0.98), Oshadha (*r*^2^ = 0.98), Rawana (*r*^2^ = 0.96), kodo millet (*r*^2^ = 0.98), sorghum ICSV 112 (*r*^2^ = 0.96), foxtail millet (*r*^2^ = 0.99), proso millet (*r*^2^ = 0.98), and white finger millet (*r*^2^ = 0.99), exhibited dose-dependent early-stage antiglycation activity. The IC_50_ values of early-stage antiglycation activity of tested millet and sorghum samples are given in Table 1. The observed early-stage antiglycation activities were significantly (*p* < 0.05) different among the samples and the IC_50_ values ranged from 15.42 ± 0.50 to 270.03 ± 16.29 *μ*g/ml. The order of potency of samples for early-stage antiglycation activity was sweet sorghum = kodo millet = Oshadha = Rawana > sorghum ICSV 112 > proso millet = white finger millet > foxtail millet. Interestingly, sweet sorghum, kodo millet, Oshadha, and Rawana showed comparable early-stage antiglycation activity to the reference drug Rutin (IC_50_ 21.88 ± 0.15 *μ*g/ml) indicating its potential to be used in functional foods, medical foods, and nutraceutical industries.

There are extremely limited reports on early-stage antiglycation activities of millet and sorghum world-over. In those studies, the presence of antiglycation activity in millet and sorghum is explained via the presence of phenolic and other antioxidants [35, 40, 41]. A study conducted by Farrar et al. [40] showed that sorghum brans which are rich in phenolic antioxidants inhibited protein glycation reaction more prominently than sorghum brans which are low in phenolic antioxidants. Anis and Sreerama [41] reported that p-coumaric and chlorogenic acids were the main phenolic acids present in barnyard millet and they exhibited antiglycation activity via various antioxidant mechanisms. Ofosu et al. [35] have studied the antioxidant activity and antiglycation activity of millet and results showed that Finger Italian Millet which had the highest total phenolic and total flavonoid contents had the highest antiglycation activity. In the present study also, the millet and sorghum samples which showed the greatest early-stage antiglycation activities had high antioxidant properties via multiple mechanisms [22, 36]. Therefore, it is reasonable to expect that phenolics and other antioxidants could be involved in inhibiting the early-stage protein glycation reaction as free radicals play a vital role in the formation of glycated proteins. However, the exact underlying mechanisms in mediating early-stage antiglycation activities in millet and sorghum have to be investigated further. The interesting fact observed in this study is that sweet sorghum, Oshadha, Rawana, and kodo millet exhibited greater early-stage antiglycation activities compared to the reported early-stage antiglycation activities of millet and sorghum worldwide [35, 40, 41]. This might be due to the genotype, differences in the extraction protocols, and agro-climatic factors.

**Figure 4:**
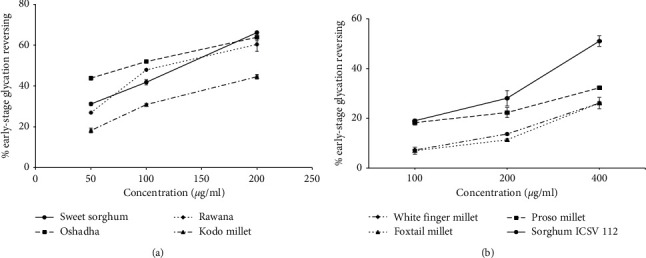
Early-stage glycation reversing activity of samples tested. (a) Dose response relationship of Oshadha, Rawana, sweet sorghum, and kodo millet. (b) Dose response relationship of sorghum ICSV 112, white finger millet, proso millet, and foxtail millet.

Reversing of already formed glycated products is an important approach in diabetes management. Thus, the compounds which can reverse the already formed glycated products might be useful as therapeutics in the management of long-term diabetes complications [7, 24, 27]. The dose response relationship of early-stage glycation reversing activity of selected millet types and sorghum varieties in Sri Lanka is shown in Figures 4(a) and 4(b). All the studied samples, namely, sweet sorghum (*r*^2^ = 0.99), Oshadha (*r*^2^ = 0.99), Rawana (*r*^2^ = 0.98), kodo millet (*r*^2^ = 0.99), sorghum ICSV 112 (*r*^2^ = 0.99), proso millet (*r*^2^ = 0.97), foxtail millet (*r*^2^ = 0.98), and white finger millet (*r*^2^ = 1), exhibited significant (*p* < 0.05) and dose-dependent glycation reversing activities. The EC_50_ values of early-stage glycation reversing activity of tested millet and sorghum samples are given in Table 1 and the EC_50_ values ranged from 91.82 ± 6.56–783.20 ± 61.70 *μ*g/ml. The order of potency of selected millet types and sorghum varieties for early-stage glycation reversing was Oshadha = Rawana = sweet sorghum > kodo millet > sorghum ICSV 112 > white finger millet = proso millet = foxtail millet. Out of the samples studied, Oshadha, Rawana, and sweet sorghum showed significantly high (*p* < 0.05) early-stage glycation reversing activity compared to the other samples tested. It is interesting to note that the order of potency of early-stage glycation reversing activity of millet types and sorghum varieties tested in the present study is different from the early-stage antiglycation activity indicating that different compounds in millets and sorghum might be responsible for the observed differences. There are no previous reports on glycation reversing activity of millets and sorghum world-over thereby comparison make impossible. Further, there are no clinical drugs approved to date as glycation reversing/cross link breaking agents [7]. This strongly suggests the importance of discovering novel glycation reversing agents/AGEs cross link breakers as therapeutics in the management of diabetes and its deliberate complications. Therefore, findings of this study are vital as these millets and sorghum may have the potential in utilizing in functional food formulations in the management of diabetes complications.

#### 3.2.2. Middle-Stage Antiglycation and Glycation Reversing Activities

**Figure 5:**
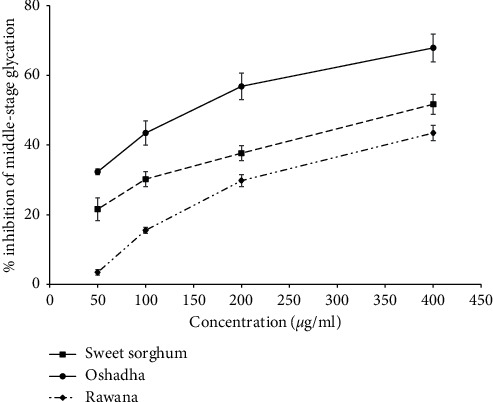
Dose response relationship of middle-stage antiglycation activity of tested samples.

Middle-stage protein glycation activity was studied via BSA-MGO model. BSA was selected as it resembles to albumin in the human body and MGO is a highly reactive dicarbonyl compound produced under *in vivo* conditions during the protein glycation reaction [7, 11]. In the present study, the millets and sorghum which showed the highest early-stage antiglycation activity were studied for middle-stage antiglycation activity and the results are given in Figure 5. Further, IC_50_ values of middle-stage antiglycation activity of tested millet and sorghum samples are given in Table 1. All three studied samples, namely, sweet sorghum (*r*^2^ = 0.99), Oshadha (*r*^2^ = 0.98), and Rawana (*r*^2^ = 0.99), showed significant (*p* < 0.05) and dose-dependent middle-stage antiglycation activity. The IC_50_ values of these samples ranged from 135.08 ± 12.95–614.54 ± 6.99 *μ*g/ml. The order of potency of samples was sweet sorghum > Oshadha > Rawana. The observed middle-stage antiglycation activities were moderate compared to the reference standard Rutin (IC_50_ 63.36 ± 0.67 *μ*g/ml) used in this study.

Studies on middle-stage antiglycation activity of millets and sorghum are extremely limited. To the best of our knowledge, there are two reported studies on BSA-MGO mediated middle-stage antiglycation activities of different finger millet and sorghum varieties world over [40, 42]. Results of the above stated studies have shown that pigmented millet and sorghum varieties had the highest middle-stage antiglycation activities. In the present study, we observed the highest middle-stage antiglycation activities in pigmented millet types and sorghum varieties (sweet sorghum, Oshadha, and Rawana) and therefore our findings are also in accordance with their findings. However, reported studies [40, 42] have not focused on the dose-dependent mechanisms and therefore comparison is impossible. The observed middle-stage antiglycation activities of millet and sorghum varieties cultivated in Sri Lanka may be due to the presence of high amounts of phenolic and other antioxidants [22, 36] as antioxidants can act as glycation inhibitors. However, the exact mechanisms could not conclude at this point due to lack of research conducted on this topic to date.

**Figure 6:**
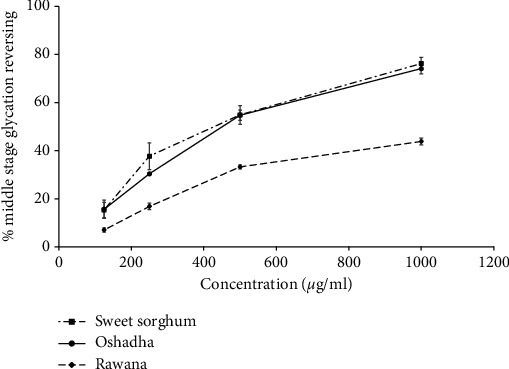
Middle-stage glycation reversing activity of samples tested.

Middle-stage glycation reversing agents can be used as therapeutics in the management of long-term diabetes complications [7]. The varieties which showed the highest early-stage glycation reversing activity were studied for middle-stage glycation reversing activity and results are given in Figure 6. Further, the EC_50_ values of middle-stage glycation reversing activity of tested millet and sorghum samples are given in Table 1. The three samples studied, namely, sweet sorghum (*r*^2^ = 0.99), Oshadha (*r*^2^ = 0.99), and Rawana (*r*^2^ = 0.99), exhibited significant (*p* < 0.05) and dose-dependent middle-stage glycation reversing activity. EC_50_ values of samples ranged from 393.24 ± 8.68–1374.60 ± 129.30 *μ*g/ml and Sweet sorghum and Oshadha showed the highest activities. The order of potency of tested samples was sweet sorghum = Oshadha > Rawana. In comparison of the findings of the early- and middle-stage glycation reversing activities of samples studied, early-stage glycation reversing is significantly high in tested millet and sorghum samples compared to the middle-stage glycation reversing indicating that different compounds and/or their efficacy might play a role in mediating the observed differences. In the absence of middle-stage glycation reversing clinical drugs, findings of this research inevitably added value to the cereal industry and especially for minor cereals, which have not yet received much attention in the cereal world.

## 4. Conclusion

Results of the present study showed that selected millet types and sorghum varieties in Sri Lanka exhibited range of health food properties in relation to management of diabetes and its complications. The observed biological activities included inhibition of *α*-amylase (a key enzyme involve in starch digestion), inhibition of both early- and middle-stage protein glycation, and reversing of both early- and middle-stage glycation products. Among the studied millet and sorghum samples, the pigmented samples, namely, sweet sorghum, Oshadha, and Rawana, showed the highest antiamylase, antiglycation, and glycation reversing activities compared to the other samples tested. Therefore, consumption of whole grains of especially pigmented millets and sorghum varieties in Sri Lanka may play an important role in the prevention and management of diabetes and its complications. Further, they can be used in functional foods and nutraceutical industries in developing value-added products. Furthermore, samples which showed potent activities may be potential natural sources for development of promising novel drugs. However, the exact mechanisms and efficacy *in vivo* should be evaluated in future research studies.

## Figures and Tables

**Table 1:** IC_50_ and EC_50_ values of millet and sorghum samples for tested bioactivities.

Millet type/sorghum variety	Activity				
	Antiamylase activity (IC_50_: *μ*g/mL)	Early-stage antiglycation activity (IC_50_: *μ*g/mL)	Early-stage glycation reversing activity (EC_50_: *μ*g/mL)	Middle-stage antiglycation activity (IC_50_: *μ*g/mL)	Middle-stage glycation reversing activity (EC_50_: *μ*g/mL)
Sweet sorghum	33.34 ± 1.11^a^	15.42 ± 0.50^a^	131.91 ± 1.95^a^	135.08 ± 12.95^a^	393.24 ± 8.68^a^
Oshadha	430.90 ± 33.50^b^	28.13 ± 1.37^a^	91.82 ± 6.56^a^	356.97 ± 22.4^b^	451.8 ± 24.2^a^
Rawana	1446.70 ± 54.10^c^	41.14 ± 1.08^a^	122.33 ± 3.72^a^	614.54 ± 6.99^c^	1374.60 ± 129.30^b^
Kodo millet		22.81 ± 1.71^a^	227.68 ± 10.21^b^		
Sorghum ICSV 112		107.27 ± 1.73^b^	394.36 ± 9.81^c^		
Proso millet		240.5 ± 25^c^	781.20 ± 33.3^d^		
White finger millet		242.87 ± 7.19^cd^	779.87 ± 4.75^d^		
Foxtail millet		270.03 ± 16.29^d^	783.20 ± 61.70^d^		

Results are expressed as mean ± SD, *n* = 3. Mean IC_50_ and EC_50_ values in columns superscripted by different letters are significantly different at *p* < 0.05.

## Data Availability

Data for the current study are available from the corresponding author upon reasonable request.
